# Modulating expression of inhibitory and stimulatory immune ‘checkpoints’ using nanoparticulate-assisted nucleic acid delivery

**DOI:** 10.1016/j.ebiom.2021.103624

**Published:** 2021-10-20

**Authors:** Adam A Walters, Baljevan Dhadwar, Khuloud T. Al-Jamal

**Affiliations:** Institute of Pharmaceutical Science, Faculty of Life Sciences & Medicine, King's College London, Franklin-Wilkins Building, 150 Stamford Street, London SE1 9NH, United Kingdom

**Keywords:** Immune checkpoint, Nanoparticle, RNA interference, siRNA

## Abstract

Immune checkpoints are regulatory molecules responsible for determining the magnitude and nature of the immune response. The aim of immune checkpoint targeting immunotherapy is to manipulate these interactions, engaging the immune system in treatment of cancer. Clinically, the use of monoclonal antibodies to block immunosuppressive interactions has proven itself to be a highly effective immunotherapeutic intervention. Within the literature there are numerous candidates for next generation of immune checkpoint targeting strategies. One such example is the use of nucleic acid to alter expression levels of immune checkpoint molecules, either as antisense oligo nucleotides/siRNA, to downregulate inhibitory molecules, or mRNA/DNA, to express co-stimulatory molecules. A significant component of nucleic acid delivery is its formulation within a nanoparticulate system. In this review we discuss the progress of the preclinical application of nucleic acid-based immunotherapies to target a selection of co-inhibitory/co-stimulatory molecules. Furthermore, we identify the potential and current gaps within the literature which may form the basis of future work.

## 1 Introduction

Immunotherapy is a well-established field of cancer treatment based on utilising the immune system to fight cancerous cells. There are currently 3 types of T cell-based immunotherapy: active vaccination, adoptive cell transfer therapy and immune checkpoint blockade. Active vaccination uses tumor antigens to induce antitumor immunity, whilst adoptive cell transfer infuses autologous lymphocytes that can be genetically engineered to respond to antigens specifically expressed on tumor cells [1]. This review will focus on the third type: immune checkpoint blockade. Immune checkpoints are molecules that regulate immune pathways to protect against autoimmunity and control the extent and duration of immune responses, preventing damage from excessive immune activation. In cancerous conditions these receptor interactions dampen an effective anti-tumor immune response, furthermore cancer cells may hijack these axes to subvert an immune attack [2]. Immune checkpoint blockade therefore aims to block these immunosuppressive interactions to allow an effective immune response. This approach has proven to be hugely successful as it overcomes many of the negative side effects associated with traditional cancer treatments such as systemic toxicity, lack of specificity and cancer drug resistance [3]. Monoclonal antibodies (mAbs) are the most well-established means to deliver immune checkpoint blockade and have proved highly successful [4].

Currently antibodies targeting cytotoxic T lymphocyte associated protein 4 (CTLA4) and the programmed cell death protein 1 and its ligand (PD-1, PD-L1) are utilised clinically, and T cell modulators are indicated for treatment of c.50 cancer types [5]. Immune signalling molecules can be divided into: inhibitory, suppressing the immune response, or more recently described, stimulatory, stimulating the immune response [6]. While antibodies targeting inhibitory immune checkpoints are well established, antibodies targeting stimulatory molecules have yet to reach clinical translation though they have been the subject of several trials [7]. Should these targets prove valid, either used in isolation or in combination with conventional immune checkpoint blockade, they may represent the of future cancer immunotherapy [8].

**Fig. 1:**
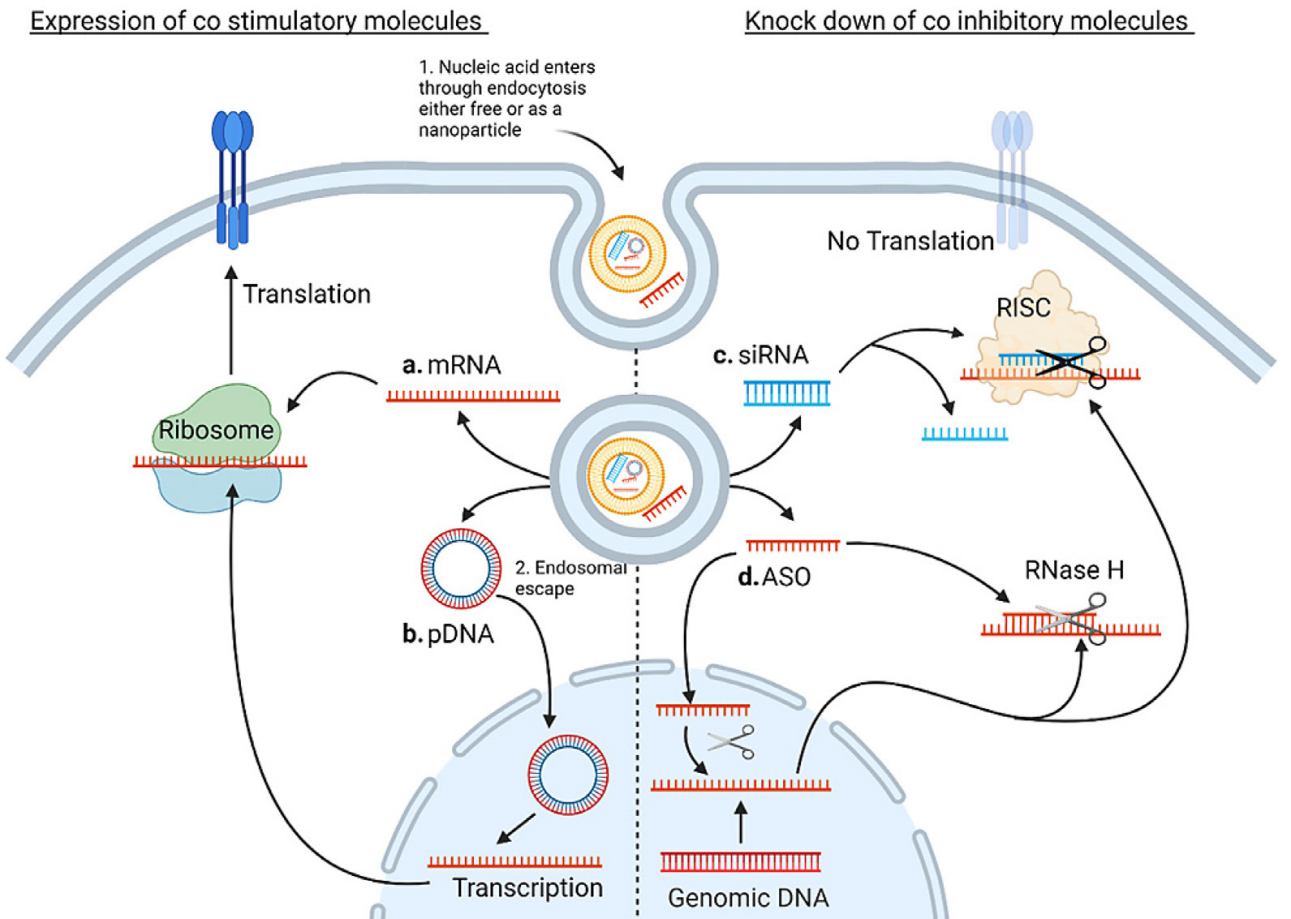
Summary of the mechanisms of the molecules discussed in this review. Nucleic acid, either as a free molecule or as a nanoparticle, first enters the cell, commonly through endocytosis. The drug must then escape the endosome before traversing to the site of action. a. mRNA is transferred to the ribosome where it is translated to protein b. pDNA must first translocate to the nucleus where it is transcribed to mRNA before being translated to protein on the ribosome. A single pDNA molecule can produce many transcripts however trafficking to the nucleus is a significant hurdle c. The guide strand of the siRNA duplex, complementary to the target mRNA, becomes associated with the RNA-Induced Silencing Complex (RISC). The RISC complex scans the mRNA for the complementary sequences and cleaves the mRNA d. ASOs can be active in both the cytosol and the nucleus. They have numerous mechanisms of action including steric blocking, modulation of intron splicing and engagement of RNAse H all of which prevent the successful translation of target protein. Original image drawn using Biorender.

While the monoclonal antibody platform has shown itself robust, there is a growing body of preclinical literature assessing alternates to monoclonal antibodies. One such approach is to use nucleic acid; nucleic acid may be a suitable substitute for the monoclonal platform as it can both downregulate immunosuppressive molecules, as siRNA or antisense oligo nucleotides (ASO), and express co-stimulatory molecules, as mRNA or plasmid DNA (pDNA). The mechanism of each of these molecules is summarised in Fig. 1. In addition to these molecules immune checkpoint expression may also be modified with the use of miRNAs, these will not be discussed in this review as they have been reviewed elsewhere and can involve a complex interplay between multiple immune modulatory pathways having both direct and indirect actions on immune checkpoint expression [9], [10], [11].

Nucleic acid is typically delivered in a nanoparticulate system, which can be divided broadly into the following categories: polymeric, lipidic or inorganic (including metallic) [12]. The minimal requirements of a particulate system are to condense the nucleic acid, allow passage through the cell membrane, and facilitate endosomal escape. In addition, the carrier must also have an element of cellular targeting, whether passive or active, to ensure some degree of selective transfection [13].

The major advantage of nucleic acid over monoclonal antibodies is that nucleic acid is relatively cheap and easy to produce [8]. Small nucleic acid constructs can be made entirely chemically, and mRNA can be produced in cell-free *in vitro* transcription reactions in large quantities. Because of this it has been suggested that mRNA encoding monoclonal antibodies may represent the next step in the evolution of the field [14].

In addition, it may be speculated that, nucleic acid may afford several theoretical pharmacological/biological advantages over traditional monoclonal antibody systems in the immunotherapy setting including:

• The ability to easily co-formulate multiple nucleic acids/chemotherapeutic drugs/immune active compounds in a single nanoparticulate system ensures co-delivery to cells or physiologic compartments in a spacio-temporally restricted manner.
• Nucleic acid can be used to target intracellular molecules such as enzymes or transcription factors which are typically inaccessible to antibodies.
• The use of monoclonal antibodies is associated with a range of potentially fatal adverse effects including colitis, pneumonitis, and hepatitis caused by excessive immune activation [15,16]. The nanoparticulate systems carrying nucleic acid may be targeted to the tumor *via* surface moieties. This can reduce off target effects and localise checkpoint blockade at the tumor site. This may be advantageous if the target molecule is widely expressed on non-cancerous cells, for example CD47 (see later section).
• The nucleic acid platform has the potential to be extremely versatile. Formulations, once developed, can be readily personalised, incorporating several nucleic acid molecules according to the patient requirements. Nucleic acid molecules may be swapped in or out of the formulation based on the progression of the disease or perceived clinical benefit. Furthermore, novel combinations of checkpoint inhibitors can be trialled in a comparatively high throughput manner.

**Fig. 2:**
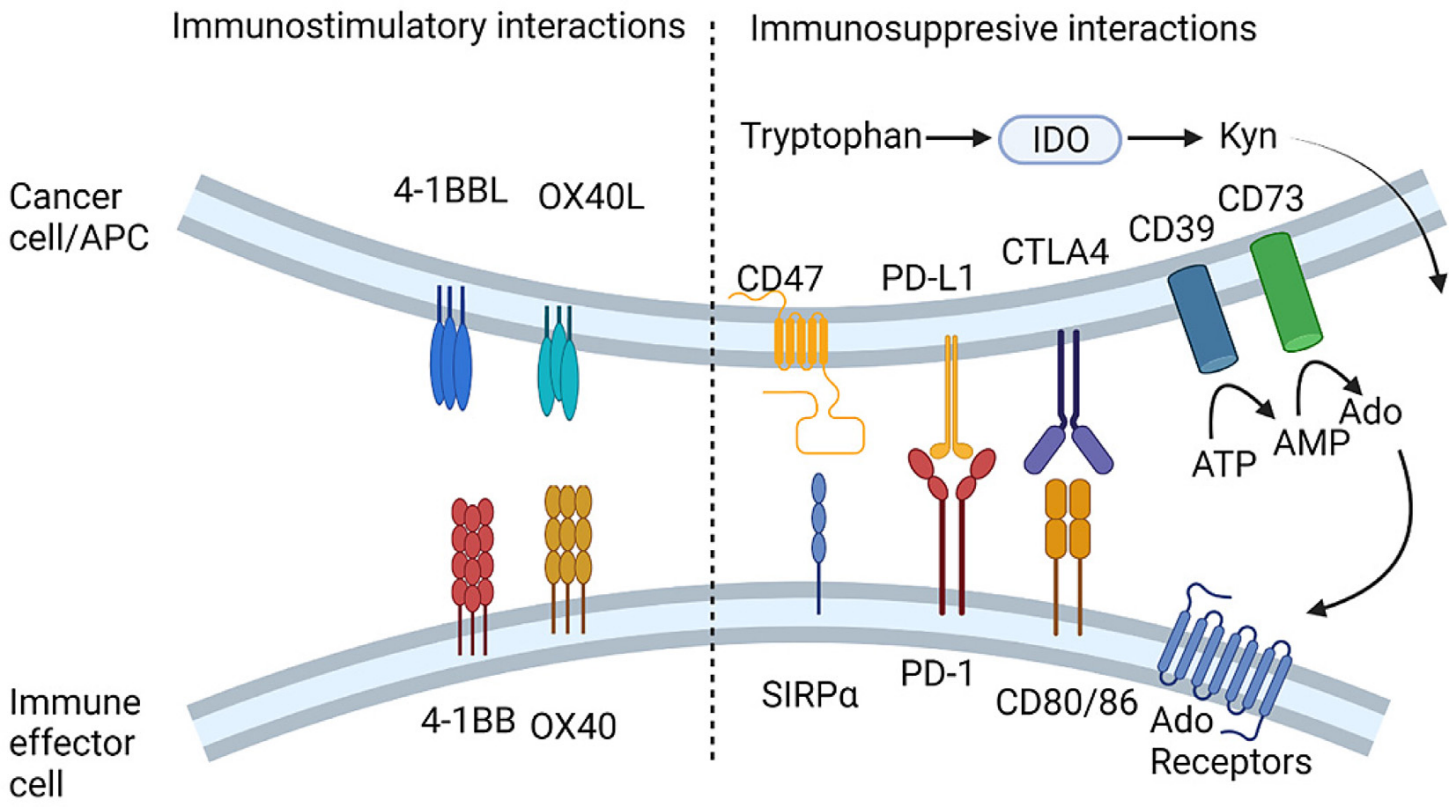
Summary of the immune targets discussed in this review. Abbr.: Ado, Adenosine; AMP. Adenosine monophosphate; ATP, Adenosine triphosphate; APC, antigen presenting cell; CTLA4, Cytotoxic T lymphocyte Associated protein 4; IDO, Indoleamine 2,3-dioxygenase; Kyn, kynurenins; PD-1/PD-L1, Programmed Cell Death Protein-1/Ligand 1 SIRPα, Signal Regulatory Protein α. Original image drawn using Biorender.

This review will focus on the work performed using nucleic acid to alter expression of co-inhibitory/stimulatory molecules with examples of the molecular target (as illustrated in Fig 2.). Targets will be segregated based on whether they are considered stimulatory, which studies aim to upregulate, or immunosuppressive, which studies aim to downregulate. Genetic modification of cell-based therapies, such as dendritic cell vaccines, will not be covered beyond providing a background. A short introduction will be provided for each target, though this should by no means be considered comprehensive. Detailed discussion of nucleic acid modification and nanocarriers has been recently reviewed elsewhere [17,18].

## 2 Using RNA to downregulate immunosuppressive targets

The use of siRNA to knock down co-inhibitory targets is the most widely employed approach to deliver nucleic acid based immune checkpoint blockade. A summary of a selection of studies which have progressed to *in vivo* testing is shown in Table 1. There is a single study identified using ASOs to target immune checkpoint molecules.

**Table 1:** Examples of *in vivo* studies conducted using nucleic acid (siRNA) to silence immunosuppressive targets.

Target	Delivery system	Physical Properties (Type /Size /Charge)	Cancer/Cellular Model	Major outcome	Route of administration	Reference	
PD-L1	Dextran coated magnetic NP	Metallic 23.2 nm	Pancreatic ductal adenocarcinoma: PAN 02	Combination treatment with gemcitabine led to 90% reduction in tumor volume after 2 weeks 100% control animals died by week 6 vs no mortality in experimental group by week 5	I.V.	[32]	
	PEI- liposomal NP	Polymer/lipid ∼160 nm +16 mV	Melanoma: B16F10	3-fold decrease in tumor volume compared to controls Co-delivery showed significantly lower tumor growth rate (0.03 compared to 0.12 of control)	I.V.	[33]	
	Fluorinated polymerised and paclitaxel-loaded HSA NP	Biological/polymer 150 nm +12 mV	Lung cancer: LLC Breast cancer: 4T1-Luc	Tumor inhibition, smaller and fewer metastases Increased survival from 25.5 days (control) to 55 days Increased CD8^+^ T cells, indicating increased T cell infiltration	I.V.	[24]	
	Crosslinked PEI and dermatan sulphate ternary	Polymer 200-250 nm	Melanoma: B16F10	Supressed melanoma growth, tumor specific growth rate of 0.0394 compared to 0.0796 in the control Increased IFN-γ, reflecting increased CTL activation	I.V.	[23]	
	Reactive oxygen species responsive chitosan NP	Polymer 139-142.7 nm +27.3 mV	Breast cancer: 4T1	Co-delivery of NPs with doxorubicin showed stronger antitumor response due to T7 targeting Co-delivery showed the highest PD-L1 downregulation	I.V.	[73]	
	PEI-based NP	Polymer 50 nm	Ovarian carcinoma: ID8-Luc	PD-L1 silencing increased T cell expansion and number of tumor-specific CD8^+^ T cells resulting in increase in survival Non targeting siRNA and PEI alone activated TLRs	I.P	[20]	
	Tumor-targeted lipid-dendrimer-CaP NP	Inorganic 110.5 nm -7 mV	Liver cancer: HCA-1, Hep3B, JHH-7	Treatment Increased infiltration of CD8^+^ T cells and suppressed tumor growth Combination treatment with vaccine further increased survival	I.V	[37]	
PD-L1 PD-1	PLGA NP	Polymer 183.3 nm -3.62 mV	Colon cancer:MC38	Delay in tumor growth in single and co-silencing groups, with 83.3% lower tumor weight	I.V	[34]	
CTLA4	PEG and PLA lipid NP	Polymer/lipid 141.6nm +4.1 mV	Melanoma: B16	Increased CD8^+^ T cells, 40.3% vs. 18.9% control, decreased Tregs amongst tumor infiltrating lymphocytes Significantly reduced tumor growth, increased survival	I.V.	[51]	
CTLA4 PD-1	Entranster-*in vivo* transfection	Commercial reagent	Hepatoma: H22	Reduced tumor volume and weight, most significant with co-delivery (P<0.05)	I.T	[52]	
CD73	Cationic lipid nanoemulsion	Lipid emulsion 262.7nm +3.5mV	Glioblastoma:C6	60% tumor reduction Detection in rat brain 6h after nasal administration, peaked at 18h and undetectable by 32h	I.V	[74]	
	TAT-chitosan- SPIONs	Polymer/metallic 133 nm +26 mV	Colon cancer: CT26 Breast cancer: 4T1 Melanoma: B16F10	Reduced hypoxia-induced angiogenesis Reduced tumor growth, most significant with simultaneous CD73 and HIF-1α suppression	I.V.	[59]	
	Chitosan lactate NP	Polymer	Breast cancer: 4T1	Tumor regression and Increased survival time Decreased angiogenesis promoting factors	I.V.	[75]	
CD47	Liposome-protamine-hyaluronic acid NP	Lipid/biological ∼70 nm +20 mV	Melanoma: B1610	Inhibited growth of tumors by ∼93% (P<0.0001) Inhibited lung metastasis to ∼27% of the untreated control and were smaller	I.V.	[62]	
	Glutamine-functionalized branched PEI	Polymer 96 nm ∼+26 mV	Lung cancer: A549 and HLF cell lines	Glutamine modified carrier decreased tumor growth the most (P<0.001), further decreased by combination treatment with cisplatin	I.V.	[63]	
	Coreshell-corona polyion complex NP	217.47-257.1 nm -3.38-+6.79mV	Breast cancer:4T1	Co-delivery of CD47 and CCL25 increased CD8^+^ tumor infiltrating T cells, reduced tumor growth rate and suppressed metastasis	I.V	[64]	
CD47 PD-L1	EpCAM- targeted cationic liposome NP	Lipid 171.7nm +31.7mV	Lung cancer: PC-9 Breast cancer: 4T1	Co-silencing decreased tumor growth by 87% and metastasis by ∼85%. Co-silencing inhibited tumor growth more than single gene silencing	S.C	[36]	
IDO	Lipid NP	Lipid 155nm +5.1 mV	Lymphoma: E.G7-OVA	Significant inhibition of tumor growth after only 12 days IDO silenced bone marrow-derived cells enhanced the antitumor effect	I.V.	[69]	
	DNA plasmid	Plasmid delivered by gene gun	Bladder cancer: MBT-2 Colon cancer: CT26	Inhibited tumor growth and prolonged survival (P=0.003) in both cancer models Adoptive transfer of CD11c^+^ cells from IDO vaccinated mice delayed MBT-2 tumor progression	S.C	[72]	
	Gold nanorods	Metallic	Lung Cancer: LLC	Combination therapy of siIDO and laser irradiation most significantly reduced tumor growth by day 22	I.V	[70]	
	MgAl-layered double hydroxide NP	Inorganic 295.3-396.1 nm +28.5-+35.5mV	Melanoma: B16F10	siIDO significantly inhibited tumor growth but most inhibition occurred with combination treatment with Trp2	S.C.	[71]	

Abbr.: NP, nanoparticle; PEI, polyethyleneimine; PLGA, poly lactic glycolic acid; HSA, human serum albumin; mV, millivolts; nm, nanometres; CaP, Calcium phosphate; I.V., intravenous; I.P., intraperitoneal; S.C., subcutaneous.

### 2.1 PD-L1 & PD-1

PD-1 is expressed on activated T cells while PD-L1 is expressed primarily on antigen presenting cells (APCs) (such as dendritic cells, DC). However, it is also over expressed in some cancers as an immune escape mechanism. PD-1, PD-L1 interactions serve to dampen the T cell response resulting in T cell anergy [19]. The use of siRNA to silence PD-L1 is the most widely reported nucleic acid based immune checkpoint targeting immunotherapy.

In early studies it was demonstrated that a relatively simple formulation of siPD-L1 combined with cationic transfection reagent (polyethylenimine, PEI) could result in T cell expansion and a high degree of survival following tumor challenge [20]. Furthermore, that the combination of non-specific siRNA and PEI displayed potent antitumor effects *via* DC activation and stimulation of multiple toll like receptors (TLRs) [20]. However, this effect was not as pronounced as the siPD-L1 construct which was able to induce tumor-specific memory CD8^+^ T cells. Since, PEI has been utilised in many siPD-L1 formulations and the backbone has been improved upon through the addition of targeting moieties such as folic acid and dermatan sulphate [21], [22], [23]. A study by Li *et al*. likewise demonstrated that PEI formulated siPD-L1 could prolong survival in a lung cancer model, however, they also showed that a rationally formulated albumin based nanocomplex (FX/HP) comprising paclitaxel, a fluorinated CXCR4 antagonist and siPD-L1 were significantly superior [24]. In addition to delivering siRNA, the FX/HP nanoparticles were immunogenically active, able to induce DC maturation and antagonise CXCR4 thus improving T cell infiltration [24]. This is particularly useful as tumors may be resistant to PD-L1 blockade as the fibrotic tumor microenvironment prevents T cell infiltration.

This study highlights the potential of siPD-L1 and carrier to be formulated to obtain synergistic or additive effects which would otherwise be hard to achieve with a traditional antibody-based formulation. Combination of siPD-L1 with chemotherapy has been attempted by several groups. The particulate nature of the siRNA carriers lends itself to co-formulation with drugs, as many of the carriers were originally developed, or have been extensively used, in the drug delivery field. A good example of this can be seen in co-formulation of siPD-L1 with anthracycline drug doxorubicin (Dox). A liposomal formulation of Dox (Doxil) is currently utilised clinically and therefore represents a suitable candidate for particulate formulation [25]. In addition, Dox has also been demonstrated to induce immunogenic cell death (ICD), a form of apoptosis resulting in the release of immunostimulatory factors (including acellular ATP and HMGB1) and translocation of calreticulin to the cell surface, therefore co-formulation with siPD-L1 may be logically justified [26]. A number of recent studies have formulated Dox with siPD-L1 utilising polymeric or lipid polymer hybrid systems [27], [28], [29], [30], [31]. In each case it has been demonstrated that the combination of Dox with siPD-L1 results in reduced tumor growth compared to monoformulated drug. Notably, Wang *et al*. formulated siPD-L1 with Dox in a self-assembling lipid polymer hybrid nanoparticle and demonstrated a 30% clearance of tumors following treatment [28]. To improve tumor targeting of siPD-L1/Dox formulations, particles have been developed which are sensitive to various aspects of the tumor microenviroment including pH, reactive oxygen species or reduction [30,31]. Particles may also be targeted *via* surface ligands such as T7 peptide which bind to the transferrin receptor overexpressed on tumor cells [31].

Drug/siPD-L1 synergy is, however, not limited to ICD inducers, for example Yoo *et al*. combined siPD-L1 with gemcitabine, a drug not typically associated with ICD, in a magnetic nanoparticle system [32]. In a pancreatic cancer model, the drug combination treatment reduced cancer cell proliferation, leading to decreased tumor growth and increased survival rates, this was particularly apparent when using high doses, with 67% of mice surviving to the end of the study (all control mice were euthanised on week 6). The prognosis for pancreatic cancer is extremely poor with a 1% 5-year survival rate and tumors are typically refractory to conventional immune checkpoint blockade [32]. Novel, rationally formulated therapies such as nucleic acid-based immune checkpoint blockade may provide some hope in the development of future therapeutics. In a similar vein, Li *et al*. combined siPD-L1 with imatinib in a liposomal system, this formulation significantly downregulated PD-L1 expression which in turn was linked to tumorigenic mTOR pathway inhibition both *in vitro* and *in vivo* [33]. In their model, PD-L1 silencing sensitised cancer cells to imatinib, resulting in higher apoptosis and therefore decreased tumor volume and growth rate. Immunogenically, the combination of imatinib cytotoxicity, mTOR pathway inhibition and PD-L1 silencing synergistically enhanced IFN-γ production [33]. The interplay between PD-L1 silencing and mTOR autophagy has been utilised in a number of studies [34].

In addtion to siPD-L1 drug combinations, siRNA may also be combined with other nucleic acid constructs such as other siRNAs, plasmid DNA (pDNA) or mRNA [35], [36], [37]. In one notable example, siPD-L1 was combined with a pDNA construct expressing IL-2 in a single lipid-dendrimer-calcium phosphate formulation [37]. IL-2 promotes T cell proliferation and enhances effector T cell activity, but requires repetitive administration which can cause negative side effects [37]. In this study, the combination of the two nucleic acid constructs enhanced antitumor responses through increased cytotoxic T cell proliferation and CD8^+^ T cell infiltration resulting in reduced tumor growth and increased survival rates [37]. It is worth considering that siRNA and pDNA have distinct spatial requirements, the siRNA being active in the cytosol and the pDNA needing to reach the nucleus. The developed formulation looks to be a promising candidate for potent co-delivery of pDNA and siRNA, though the exact mechanisms and intracellular trafficing of pDNA/siRNA molecules was not established. In contrast to pDNA, mRNA is active in the cytosol and therefore may represent a more logical choice for co-formulation with siRNA. Indeed, the inclusion of siPD-L1 into an mRNA cancer vaccine delivered *via* lipid coated calcium phosphate nanoparticles was shown to reduce tumor growth and enhance IFNγ responses [35]. Interestingly, this study demonstrated the inclusion of siPD-L1 was more effective than the co-delivery of anti PD-L1 mAbs [35]. While there have been few studies comparing siRNA to mAb, those which have typically reported siPD-L1 to be more effective, whether this is reproduced clinically has yet to be demonstrated [20].

In contrast to its ligand, the silencing of PD-1 as a therapeutic intervention has been relatively understudied. This may be because there are additional difficulties targeting PD-1 as it is expressed on T cells which are generally considered a challenging target to transfect with non-viral vectors [38]. However, there are an increasing number of particulate formulations which have been developed to transfect T cells, including lipid and polymeric systems [39], [40], [41]. In many cases these technologies have been developed for the *in situ* delivery of chimeric antigen receptor expressing constructs (reviewed [42]). Platforms using anti CD3/CD4 antibodies or ScFv as a targeting moiety have achieved high *in vivo* transfection however this may undermine the aims of nucleic acid mediated immune checkpoint blockade as a means of reducing the use of monoclonal antibodies [43,44]. The use of aptamers could resolve this issue, indeed anti CTLA4 and anti 4-1BB aptamers fused to siRNA constructs have been used to target T cells *in vivo* [45,46]. These chimeric constructs may represent the future of nucleic acid immune checkpoint blockade (reviewed [47]). Alternately, platforms have been developed based on ‘constrained lipid nanoparticles’ which have demonstrated the ability to delivery siRNA to T cells *in vivo* [48].

The use of cells *ex vivo* affords the opportunity to study the relative contribution of PD-1/PD-L1 and their potential as targets for nucleic acid-based checkpoint blockade. In a candidate cell-based DC vaccine it was observed that PD-L1 silencing in DCs increased T cell proliferation and DC activation [6]. The PD-1 silencing on T cells proved even more influential in improving T cell priming, and skewing towards a Th1 phenotype, but the greatest efficacy in T cell proliferation was observed using dual silencing of both PD-1 and PD-L1 [6]. In accordance with this, it was found that silencing either PD-1 or PD-L1 increased killing of tumor cells, however co-silencing resulted in the most potent cytotoxicity [49]. In *in vivo* models, Kwak *et al*. developed a PLGA based system to deliver both siPD-L1 and siPD-1 [34]. In keeping with the *in vitro* observations, a reduced tumor growth rate was observed in both the monoformulated and co-formulated siRNAs, however the co-formulation of siPD-1 and siPD-L1 was shown to be superior with tumor growth comparable to anti PD-L1 mAb [34].

In summary, studies most commonly suppressed PD-L1 as opposed to PD-1. Combinatory treatment with other drugs or dual suppression of other checkpoint targets produced enhanced antitumor effects. However, difficulty in T cell transfection highlights the importance of a suitable carrier.

### 2.2 Cytotoxic T-lymphocyte-associated protein 4 (CTLA4)

CTLA4 was amongst the earliest immune checkpoints to be described, and it is expressed on activated T cells and Tregs. During the interaction of a T cell with an APC, CTLA4 competes with CD28 for CD80/CD86 with a higher affinity, the lack of ‘signal 2’ co-stimulation provided by this engagement results in T cell anergy [50].

Similarly to siPD-1, there are few studies on siCTLA4 potentially due to difficulty in transfecting T cells *in vivo* [38]. However, Li *et al*. developed PEG-PLA nanoparticles to deliver siCTLA4 to T cells [51]. Successful transfection was demonstrated *in vitro* and *in vivo* and, following treatment, reduced tumor volume was observed. This was marked by an increased T cell tumor infiltration and a dose-dependent increase in serum IFNγ [51]. As both PD-1 and CTLA4 are expressed by T cells, the logical combination of siPD-1 and siCTLA4 was performed by Liang *et al*. Although each siRNA construct used in isolation resulted in reduced tumor weight and volume, the largest decrease was observed using co-inhibition [52]. Co-inhibition also resulted in increased IFN-γ secretion and decreased IL-10, IL-6 and survivin [52].

### 2.3 Ecto-5’-nucleotidase (CD73) and Ectonucleoside triphosphate diphosphohydrolase-1 (CD39)

CD73 is an enzyme expressed on the surface of a number of tissues; in cancer it has been associated with increased cell proliferation, neovascularisation and tumor invasiveness [53]. As a molecule (in concert with CD39), it is responsible for the breakdown of immunostimulatory ATP to immunosuppressive adenosine [54].

There has been particular interest in silencing CD73 for the treatment of glioblastoma. It has been shown that following treatment with siCD73, there was reduced cell proliferation and glioblastoma cells cultured in the presence of adenosine had increased viability [55]. Therefore to silence CD73, Azambuja *et al*. developed cationic nanoemulsions to carry siRNA, which, upon treatment of cells, produced a large decrease in AMPase activity and cell viability [56,57]. To target glioblastoma, the optimised formulation was delivered intra-nasally to capitalise on the nose to brain-pathway. In this study it was found that nanoparticles were present in the brain six hours following adminstration and peaked at 18h before returning to undetectable levels by 32h [57]. When used in a therapy study, they observed a 60% tumor volume reduction with the siCD73 formulation with no shrinkage being observed in control goups [57].

In addition to glioblastoma, siCD73 has been trialled in breast cancer models, in a series of studies Jadidi-Niaragh *et al*. developed chitosan lactate nanoparticles which were safe, highly stable and were efficient in transfecting 4T1 triple negative breast cancer cells [58]. The base chitosan particles have subsequently been improved by the addition of a magnetic SPION core, cell penetrating peptide (TAT), drug co-formulation and/or a folate targeting moiety [59,60]. When tested *in vivo*, Ghalamfarsa *et al*. observed that silencing of CD73 resulted in decreased blood vessel formation and a reduction in angiogenic factors e.g. VEGF, which have been linked to hypoxic conditions within the tumor [59]. These treatments resulted in reduced tumor growth, cancer cell migration and colony formation [59]. Most interestingly, the anti-angiogenic effects of CD73 silencing were enhanced with co-silencing of HIF-1α which further reduces CD73 expression by reducing binding to certain cells within the hypoxic TME [59]. Together these studies illustrate the potential of siRNA when used in rationally formulated combinatory regimes.

In the only study utilising an ASO to knock down an immune checkpoint we identified, CD39 was selected as the target [61]. In this study it was reported that ASO CD39 treatment significantly increased the ratio of CD8 to Tregs, and that combination of ASO CD39 with anti PD-1 mAb resulted in reduced tumor volume [61].

### 2.4 CD47

In contrast to the other molecules described in this review which focus on engaging T cells, the exploitation of the CD47 axis primarily relies on the activity of macrophages. Though macrophage polarisation and activation will also result in augmented T cell responses. CD47 is ubiquitously expressed on several cell types but is over expressed on the cancer cell surface, it engages with SIRPα expressed on phagocytes to deliver a ‘don't eat me’ signal which prevents cell clearance through phagocytosis. As the effect of CD47 is due to macrophages, it is possible to speculate that siCD47 therapy may be most suited to cancers of organs with high resident macrophage populations.

To deliver siCD47, Wang *et al*. developed liposome-protamine-hyaluronic acid nanoparticles and treatment resulted in increased phagocytosis by macrophages, leading to reduced formation and proliferation of lung metastases [62]. A striking 93% tumor reduction was attributed to macrophages, as macrophage depletion by liposomal clondronate significantly ablated this therapeutic effect [62]. To further target siCD47 to the tumor bed, Wu *et al*. used a glutamine modified polyplex to deliver the siRNA. Cancer cells require high levels of glutamine to support their mitosis, therefore nanoparticles accumulate due to the ‘glutamine trap’ effect [63]. As a measure of this, the glutamine polyplex uptake was low in healthy cells but was triggered by glutamine deprivation as a result of cancer cells depleting the local glutamine supply. Tumor growth significantly decreased following siCD47 delivery, but more so when administered alongside the chemotherapy drug cisplatin, indicating suitability for combination treatment [63].

There have been formulations devised to deliver siCD47 whilst also enhancing T cell responses [36,64]. Indeed, siCD47 has been delivered alongside siPD-L1 in an EpCAM-targeted liposomal nanoparticle. Co-silencing of siCD47 and siPD-L1 increased IFN-γ production and, although single silencing was sufficient to reduce tumor growth, the most significant tumor growth inhibition was seen with the dual silencing nanoparticle [36]. The relative contributions of macrophages and T cells was not established though T cell numbers were elevated in formulations containing siPD-L1. To increase the numbers of CD8 T cells in the tumor Chen *et al*. developed a core shell-corona nanoparticle capable of first releasing chemokine ligand 25 (CCL25) then transfecting siCD47 [64]. CCL25 serves to inhibit CD4^+^ T cell differentiation into Treg cells and promotes survival of CD8^+^ T cells [64]. The co-delivery had a range of effects including increased tumor infilitrating lymphocytes and altering CD8^+^/CD4^+^ T cell ratios and CD8^+^/Treg ratios [64]. Furthermore, tumor growth was delayed, and the size and number of metastases decreased. siCD47 monotherapy only slightly inhibited tumor growth and CCL25 monotherapy had no effect on tumor growth [64]. Interestingly, the antitumor effects were inhibited in CD8^+^ depleted mice, demonstrating that antitumor efficacy was CD8^+^ T cell dependent [64].

### 2.5 Indoleamine 2,3-dioxygenase 1 (IDO)

IDO is an intracelluarly expressed enzyme, expressed by antigen presenting cells and cancer cells, it is responsible for converting tryptophan to kynurenine [65]. Kynuenine and tryptophan starvation has various downstream effects on T cells, including the suppression of proliferation and the induction of Treg functions [66]. As an intracellular target it is typically inaccessible to antibodies and pharmacological inhibition is typically performed using small molecule inhibitors [67].

The use of siRNA to silence IDO has the potential to be more potent or comparable to small molecule inhibition. Zheng *et al*. demonstrated that silencing IDO in cancer cells prior to implantation resulted in significantly delayed tumor growth and was superior to small molecule inhibitor L-1-Methyltryptophan (1-MT) [68]. When used as a therapeutic intervention, the liposomally formulated siIDO expressing plasmid resulted in reduced tumor growth [68]. Rather than silencing the cancer cells, Endo *et al.* investigated siIDO silencing in dendritic cells, following implantation of cancer cells and subsequent treatment with IDO silenced denditic cells reduced tumor growth was observed [69].

To develop an siIDO platform for *in vivo* testing Zhang *et al*. developed a folate targeted, gold nanorod based system, for combined siRNA delivery and photothermal therapy [70]. As photothermal therapy elevates the level of IDO, it was speculated that the combination with IDO silencing would result in synergistic effects. When tested, it was demonstrated that the combination of siIDO with photothermal therapy resulted in significantly reduced tumor growth, an increase in CD4^+^ and CD8^+^ tumor infiltrating lymphocytes, reduced T cell apoptosis and increased TNF-α and IFN-γ [70]. It was also observed that siIDO, when used in isolation, resulted in a remarkable reduction of tumor growth. [70] The positive effects of siIDO were also observed when delivered by MgAl hydroxide nanoparticles [71]. When combined with Trp2, a melanoma-associated antigen, it was demonstrated that siIDO resulted in tumor growth reduction, increased CTL activity and elevated levels of serum IFN-γ [71]. The authors speculated this was due to presentation of the peptide by the DC whilst the siIDO removed immunological inhibition [71].

Dendritic cells were further targeted in a study by Yen *et al*. who used a biolistic device to deliver siIDO to the skin, an area particularly rich in dendritic cells. In this model they observed reduced tumor growth and prolonged survival in siIDO treated mice, notably siIDO treatment improved survival compared to systemic long term 1-MT administration [72]. When studying the mechanisms of protection, it was observed that adoptive transfer of CD11c^+^ cells (DCs) from siIDO treated mice into tumor bearing mice delayed tumor progression, and also that depletion of CD8 T cells abrogated tumor regression [72].

Combined, these studies demonstrate that siIDO is a viable candidate for siRNA-based therapy, particularly when dendritic cells are the primary targets. The IDO homologue: IDO2, has also been targeted with siRNA though the effects were less pronounced [72]

## 3 Using mRNA/pDNA to express co-stimulatory ligands

Using nucleic acid to express co-stimulatory ligands is a relatively new concept reflective of the larger immune checkpoint field. In many cases nucleic acid expressing multiple co-stimulatory ligands or cytokines are delivered together to achieve synergistic or additive effects. What follows is a review of some of the key targets identified to date and is summarised in table 2.

**Table 2:** Examples of *in vivo* studies conducted using nucleic acid (mRNA and pDNA) to express immune checkpoint targets.

Target	Delivery system	Physical Properties (Type /Size /Charge)	Cancer/ cellular Model	Major outcome	Route of administration	Reference
OX40L CD80 CD86	Charge-altering releasable transporters (CART)	Polymer	Lymphoma: A20 Colon cancer: CT26	Total treated and distal tumor eradication with mOX40L stimulation, near-total clearance using mIL-12 or mCD80/86 Increased survival with mOX40L/mCD80/mCD86 and mOX40L/mIL-12 co-stimulation mOX40L/mCD80/mCD86 co-stimulation activated natural killer cells, CD4^+^ and CD8^+^ T cells and downregulated FOXP3 and CTLA4	I.T	[78]
OX40L IL-23 IL-36γ	Lipid NP	Lipid	Colon cancer:MC38 Hepatocellular carcinoma:H22 Melanoma: B16F10	Total tumor regression in 50% animals with single mOX40L and mIL-23 treatment (total tumor regression in all mice seen using doubled dose of IL-23 and IL-36γ) Increased recovery and survival rate with co-stimulation, further improved by triplet treatment	I.T	[79]
4-1BBL IL-12	Poly (Beta-Amino Ester) (PBAE) NP	Polymer 143 nm +23.3 mV	Melanoma: B16F10 Colon cancer: MC38	Reduced tumor growth in combination therapy (4-1BBL displayed slower growth than IL-12 in single treatment, P<0.0001) IL-12 alone and combination treatment increased tumor infiltrating leukocytes, T cells, natural killer cells Long-term survivors resisted new tumor formation	I.T	[83]

Abbr.: NP, nanoparticle; mV, millivolts; nm, nanometres; I.T., intratumoral; il, interleukin

### 3.1 OX40/OX40L

OX40 is expressed on activated T cells whilst its ligand (OX40L) is expressed on antigen presenting cells. Engagement of OX40 with OX40L serves to prolong the survival of T cells and prevent the differentiation of CD4 T cells into Tregs [76]. It has been demonstrated that cancer cells transfected with OX40L either do not establish tumors or established very slow growing tumors [77].

When delivered as mRNA in a charge-altering releasable transporter, OX40L has been shown to be extremely potent, mice were able to eradicate 100% of tumors following intratumoral mOX40L administration, the growth of distal, non-treated tumors, was also reduced. In the same study, mOX40L was tested with mIL-12, mCD80 and mCD86 [78]. In these studies it was shown that therapy with mIL-12, mCD80 and mCD86 stimulation cleared almost all tumors with significant growth delay in distal tumors. The triple combination of mOX40L/mCD80/mCD86 demonstrated the highest efficacy in tumor regression and survival, followed by mOX40L/mIL-12, whilst mIFN-γ had no effect. The mOX40L/mCD80/mCD86 combinations led to natural killer, CD4^+^ and CD8^+^ T cell activation in draining lymph nodes, local tumors and distal tumors [78]. This was confirmed by upregulation of activation, cytotoxicity and proliferation markers. Foxp3 and CTLA4 were downregulated, further contributing to a pro-inflammatory shift in the tumor microenvironment [78]. Combinations of mOX40L with mCD70, another member of the TNF family involved in T cell stimulation, were demonstrated to have no effect [78].

In addition to co-stimulatoy molecules, the combination of mOX40L with cytokines mIL-23 and mIL-36γ was investigated by Hewitt *et al*. in a lipid nanoparticle [79]. Delivery of mOX40L resulted in total tumor regression in 50% of mice, with H22 tumors. Dual treatment with either mIL-23 or mIL-36γ prolonged survival which was further improved using triplet treatment [79]. Surprisingly, the delivery of the mRNA constructs was more potent than delivery of the recombinant proteins and resulted in >70% survival.

Immunologically, the triplet treatment increased Th1-related molecules and upregulated certain genes, indicating the cytokines IL-23 and IL-36γ were responsible for greater transcriptional changes [79]. IL-36γ was also responsible for increased CCL7 and IL-1α secretion, a later wave of IL-22, IFN-γ, TNF-α and IL-1β secretion and for the activation and proliferation of many lymphocyte types both as a single and combination treatment. Interestingly, tumors that were unresponsive to other immune checkpoint blockade treatments such as anti-PD-L1 or anti-CTLA4, were significantly reduced in size when triplet treatment was administered, results were further enhanced when used in combination with other immune checkpoint targets [79].

Combined, these studies reveal OX40L to be a highly promising candidate for co-stimulatory nucleic acid-based immune checkpoint blockade. Future work may comprise of formulating mOX40L with other drugs or immune interventions. The move away from intratumoral (I.T) delivery would also be clinically preferable and maybe achieved through the use of a targeted nanocarrier.

### 3.2 4-1BB/4-1BBL

Much like OX40 and OX40L, 4-1BB and 4-1BBL are expressed on activated T cells and antigen presenting cells, respectively. However, in contrast to OX40/OX40L interactions, which are commonly described as pertaining to CD4 T cell survival and the inhibition of regulatory function, 4-1BB/4-1BBL engagement has been shown to induce CD8 expansion [80]. For this reason it may be an attractive target for nucleic acid based immunotherapy. While there is limited work on delivering 4-1BBL as a nucleic acid construct, it has been used in DNA vaccine formulations with varying degrees of success [81,82].

To deliver 4-1BBL nucleic acid based immunotherapy Tzeng et. al. developed and screened poly(Beta-Amino ester) polymers for the co-formulation of both 4-1BBL and IL-12 plasmids. In this study it was demonstrated that intratumoral treatment with either p4-1BBL or a combination of the two plasmids resulted in long term mouse survival and what is more the dual treatment could protect from rechallenge at a distal site [83]. These effects were also observed in an alternate tumor model suggesting the formulation can induce broad, systemic immune responses [83].

## 4 Summary of nucleic acid-based checkpoint blockade and future opportunities

Investigation into immune checkpoint targets using nucleic acid-based delivery is a relatively novel area, with most research having been published in the past 5 years. The majority of this research has been on PD-1/PD-L1 due to its success with mAbs, however there were a number of other targets identified in this review that showed promising results. All studies found that treatment led to an immunostimulatory shift, whether that was through DC maturation, increased pro-inflammatory cytokines or markers of T cell activation. Most found that this resulted in reduced tumor growth and even prolonged survival. In studies using combination treatment, it was consistently found that dual or even triple treatment enhanced the antitumor effects which was the case when multiple immune checkpoints were targeted or when used in combination with chemotherapy drugs. siRNA-based delivery was most commonly used, but there was significant variation in the delivery system utilised. Polymer and lipid-based systems were most popular, probably due to accessibility and potential for downstream application. There were several interesting studies that took different approaches such as using metal nanoparticles to assist with photothermal therapy, but all systems had specific properties suited to the route of administration and cancer model.

Direct comparisons between studies are limited due to inconsistencies in approaches, materials, and cancer models. Immunotherapy and even immune checkpoint inhibitors using mAbs is ineffective in most patients due to heterogeneity between cancer types and within subtypes. Therefore, although these results appear promising it might only be effective in a subset of patients with a specific type of cancer. Moreover, despite achieving high efficacy in murine models, this may not translate as effectively in human patients. There was further variation amongst the studies, including the target, route of administration, dose, delivery system, nucleic acid, sample size and outcomes measured.

There are multiple molecules in the pipeline being investigated as immune checkpoint targets, however, to date many have not been explored as nucleic acid-based delivery. For example, human leukocyte antigen G (HLA-G) which provides immunity to foetuses in pregnant women but has been reported to be overexpressed in many tumor types [84]. As highlighted by siIDO, nucleic acid checkpoint blockade is able to silence intracellularly localised molecules, there is, therefore, the prospect of silencing immunosuppressive regulatory or transcription factors. Indeed, work has been published silencing SOCS1 and STAT3, this may be an exciting area of future research [45,85]

Several studies highlight the efficacy of combinatory treatments, therefore research into future targets could exploit dual targeting by selecting specific combinations of targets based on the target cell or effect. Examples of synergistic silencing may include: co-silencing PD-1 and CTLA4, which are both expressed by T cells [52]. Silencing of CD47 to remove the ‘don't eat me signal’ whilst also silencing CD73 on tumor to increase the accumulation of anti-tumorgenic M1 macrophages [86]. Individually, siIDO and mCD40 prolong mouse survival when delivered to DCs, there is, therefore, potential to combine these two molecules into a single formulation [87]. A similar approach can be utilised when selecting drug-nucleic acid combinations. For example: as several chemotherapeutics are known to cause the release of ATP, it may be logical to co-formulate these with siCD73/siCD39 to potentiate the immunostimulatory effect [88].

Using drugs to target co-stimulatory checkpoints is still a relatively novel concept which is likely to grow in popularity in the coming years. Furthermore, with mRNA-based vaccines being recently licenced for COVID-19, it is likely the clinical acceptability of these technologies will cause their prevalence to increase. The advent of this technology will enable many more co-stimulatory targets to be assessed, including CD70, ICOS and GITRL.

With regards to formulation, it has been shown that there is considerable variation between cancer cell lines in terms of transfection efficiency [89]. It may be predicted that this diversity will be reflective of clinical disease. In future, the development and comprehensive testing of formulations suitable for transfecting multiple cell lines, including difficult to transfect lines, will greatly progress our understanding of the limitations and obstacles to nucleic acid delivery. Within the scientific community, there is an ongoing debate with regards to validity of using the enhanced permeation and retention (EPR) effect to deliver nanoparticles to tumors clinically [90]. Developing nanoparticles which transfect the tumor selectively, either through addition of targeting ligands, modification of nanoparticle properties or direct intratumoral injection would greatly enhance their potential. Furthermore, development of formulations which can reliably and selectively target T cells with high efficiency *in vivo* would allow improved silencing of T cell expressed targets. In contrast to cancer cells, the transfection efficiencies of T cells are likely to be more consistent between patients and may be seen as prime target for nucleic acid immune checkpoint blockade as they express multiple regulatory molecules, for example, T-cell immunoglobulin mucin-3 (TIM-3) and lymphocyte activation gene 3 (LAG-3) [91,92]. In conclusion, there have been numerous preclinical studies assessing nucleic acid as a replacement for monoclonal antibody-based therapeutics. In all studies assessed, the nucleic acid-based system resulted in some form of immune activation and/or tumor growth regression. As such nucleic acid is a strong candidate for next generation immune checkpoint targeting immunotherapy.

## 5 Search strategy and selection criteria

This critical review was conducted by collating studies identified from an electronic search of PubMed using the search terms; (nucleic acid) AND (immune checkpoint) AND (immunotherapy) between (01.05.20-01.09.21). Additional studies were identified using targeted Google Scholar searches with relevant key words, (for example “PD-L1” AND “siRNA”), followed by abstract and full-text screening. Studies were excluded if a nucleic acid containing formulation was not the principle therapeutic modality under investigation for example if nucleic acid was used to modify CAR-T cells.

## Declaration of competing Interest

The authors declare that there is no conflict of interest.
